# Synergistic effects of 3D chitosan‐based hybrid scaffolds and mesenchymal stem cells in orthopaedic tissue engineering

**DOI:** 10.1049/nbt2.12103

**Published:** 2023-01-28

**Authors:** Ping Qi, Zhaohui Ning, Xiuju Zhang

**Affiliations:** ^1^ Department of General Practice Linyi People's Hospital Linyi Shandong China; ^2^ Department of Traditional Chinese Medicine Taian Central Hospital Taian Shandong China

**Keywords:** bone, cartilage, Chitosan, stem cell

## Abstract

Restoration of damaged bone and cartilage tissue with biomaterial scaffolds is an area of interest in orthopaedics. Chitosan is among the low‐cost biomaterials used as scaffolds with considerable biocompability to almost every human tissue. Considerable osteoconductivity, porosity, and appropriate pore size distribution have made chitosan an appropriate scaffold for loading of stem cells and a good homing place for differentiation of stem cells to bone tissue. Moreover, the similarity of chitosan to glycosaminoglycans and its potential to be used as soft gels, which could be lasting more than 1 week in mobile chondral defects, has made chitosan a polymer of interest in repairing bone and cartilage defects. Different types of scaffolds using chitosan in combination with mesenchymal stem cells (MSCs) are discussed. MSCs are widely used in regenerative medicine because of their regenerative ability, and recent line evidence reviewed demonstrated that the combination of MSCs with a combination of chitosan with different materials, including collagen type 1, hyaluronic acid, Poly(L‐lacticacid)/gelatin/β‐tricalcium phosphate, gamma‐poly[glutamic acid] polyelectrolyte/titanium alloy, modified Poly(L‐Lactide‐co‐Epsilon‐Caprolactone), calcium phosphate, β‐glycerophosphate hydrogel/calcium phosphate cement (CPC), and CPC‐Chitosan‐RGD, can increase the efficacy of using MSCs, and chitosan‐based stem cell delivery can be a promising method in restoration of damaged bone and cartilage tissue.

## Graphical abstract

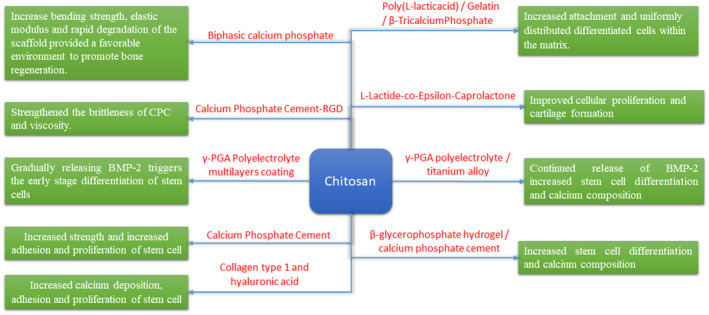

The different scaffold composition with chitosan and their effect on stem cells in the development of bone and cartilage tissue.

## 1 INTRODUCTION

### 1.1 Chitosan and orthopaedic tissue engineering

Restoration of the damaged tissue is the main focus of tissue engineering, and successful restoration of damaged tissue in orthopaedics mainly requires choosing appropriate cells and biomaterials. Biomaterials are three‐dimensional (3D) structures that act as the extracellular matrix. In many cases, the desirable biomaterial should be degradable and cytocompatible to minimise the host response and increase the dynamicity of the organ [1]. Among the natural materials used for tissue repair, polysaccharide‐ and protein‐based scaffolds are the main therapeutic choices for researchers [2]. Chitosan is a natural polysaccharide‐based scaffold consisting of deacetylated chitins as a cationic polymer, which is mainly used in the repairing of the cartilage and bone defects [3, 4]. This biomaterial interacts with surface receptors of damaged tissues and induces cell migration and improvement of extracellular production of proliferating signals. Chitosan as a biomaterial for bone and cartilage regeneration has been reported to have specific advantages and limitations as the same as every other biomaterial used for the tissue regeneration [5]. While chitosan has low toxicity and considerable biocompatibility to its environment, its biodegradability as well as controlled degradation by specific enzymes, such as lysozyme, are its main advantages as a scaffold for regenerating purposes [6]. Moreover, chitosan is able to induce the synthesis of the extracellular matrix and induce the chondrogenic activity of chondrocytes for improving the cartilage repair [7]. In the same vein, the hyaline‐like repair stimulated by chitosan‐based matrix can repair cartilage defects [8]. Moreover, the similarity of chitosan to glycosaminoglycans (GAGs) as a major component of bone extracellular matrix and its potential to be used as soft gels, which could be lasted for as long as 1 week in mobile chondral defects, has made chitosan a polymer of interest in repairing bone and cartilage defects [9]. On the other hand, chitosan has a high crystallinity because of its amino groups, making it a poorly soluble material in aqueous solutions, which is its main limitation for the clinical use [10]. However, the combination of chitosan with other molecules provides ideal properties for considering this biomaterial as a scaffold or delivery system for other biomaterials or living cells [11, 12, 13].

### 1.2 MSCs and orthopaedic tissue engineering

Stem cells are the most common cells used in regenerative medicine mainly because of their self‐renewal abilities [14, 15, 16]. Mesenchymal stem cells (MSCs) are widely used in orthopaedics diseases because of their potential in differentiation to other cell lineage [17]. MSCs are a subtype of multipotent stem cells, which are easy to isolate. This type of stem cells is distributed in different tissues, including bone marrow, blood, adipose, and human umbilical cord tissue, and is widely used in the treatment of various diseases [18, 19, 20]. Bone marrow MSCs (BM‐MSCs) and adipose‐derived MSCs are the two most common types of MSCs, which are widely used in orthopaedic diseases [17]. While there is not any remarkable difference regarding the chondrogenic and osteogenic properties of BM‐MSCs and adipose‐derived MSCs, adipose‐derived MSCs are more readily accessible in contrast to BM‐MSCs [21]. These types of stem cells have a non‐significant expression of histocompatibility complexes preventing them from rejection by the host immune system after allogenic transplantation [22]. Moreover, MSCs have homing abilities to inflammation and injured tissue. This helps them improve healing by modulating various types of chemokines and cytokines as well as secreting specific exosomes containing growth and differentiating signalling molecules [23]. During bone damage, MSCs play a major role in bone regeneration and osteogenic differentiation. After the formation of a haematoma during bone injury, immune cells are attracted to the bone lesion and an inflammatory microenviroment is developed because of the secretion of inflammatory cytokines, including IL‐6, IL‐1B, TNF‐a and IL‐17 [24]. Furthermore, MSCs with proangiogenic and osteogenic potentials secreting bone morphogenetic protein (BMP)s, TGF‐β, and vascular endothelial growth factor (VEGF) locate at the site of haematoma, proliferate, and undergo osteogenic differentiation [24]. During osteogenic differentiation and osteogenesis, the increased expression of BMPs increases the expression of osteogenic markers, including alkaline phosphatase (ALP), type I collagen, osteocalcin (OC), osteopontin (OP), osterix (OSX), and Runt‐related transcription factor 2 (Runx2) [24]. Similarly, during cartilage repair, MSCs produce and secrete various molecules, including different types of collagen, fibronectin, and GAGs, to the extracellular matrix at the inflamed cartilage sites, which are critical for cartilage functions [25]. Despite the homing potential of MSCs, there are many delivery methods introduced for better delivery of stem cells to desired sites [26, 27]. Delivery of different types of MSCs to damaged bone or cartilage tissue could be facilitated by choosing an appropriate scaffold and biomaterials [28]. To date, numerous natural and synthetic scaffolds have been introduced in the literature. Good formability of scaffolds is an important issue in tissue engineering, and the quality of these scaffolds is mainly determined by their ability to maintain or improve MSCs potential in tissue formation and producing signalling cascades [29]. Scaffolds inducing a greater release of bone morphogenic proteins as well as a high expression of bone marker ALP and growth factors, including VEGF, are generally preferable for regenerative purposes [21]. Among polymeric biomaterials, which are used as scaffolds for stem cells, chitosan is a biodegradable polymer, which is used in orthopaedic tissue engineering. The focus of the present review is evaluating the potential of chitosan‐based scaffolds for enhancing MSC‐mediated bone and cartilage tissue repair in orthopaedic tissue engineering.

## 2 CHITOSAN‐BASED HYBRID SCAFFOLDS AND ORTHOPEDIC TISSUE ENGINEERING

**FIGURE 1:**
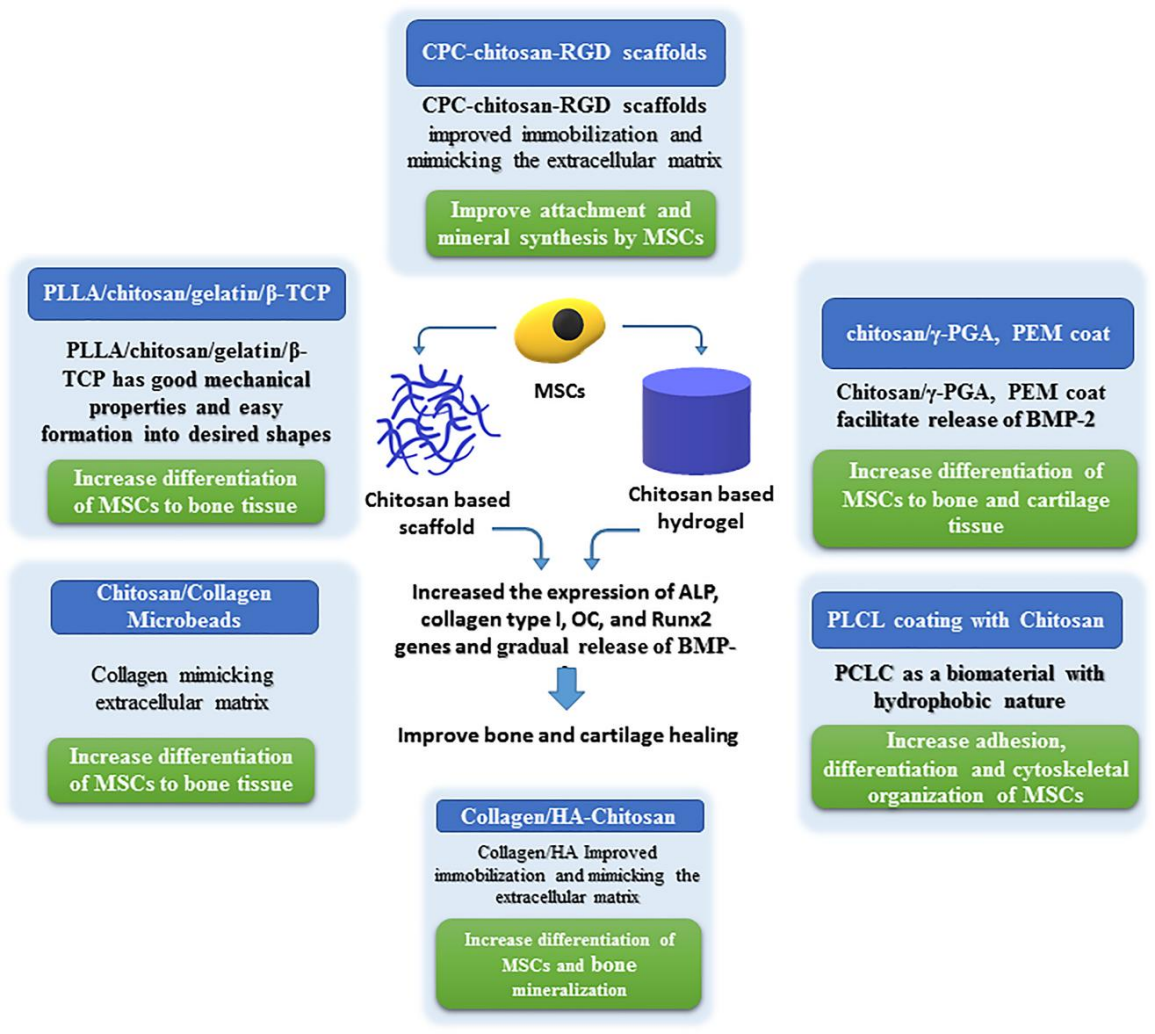
Summary of the effect of chitosan‐based materials on bone and cartilage tissue regeneration using stem cells

Chitosan is a low‐cost material, which is easy to use for scaffold processing. The biocompability of chitosan with almost all human tissues has made it a good choice for use in tissue engineering. Chitosan has been used in combination with various biomaterials to overcome their shortcomings in regenerative medicine and orthopaedic disease tissue engineering (Figure 1) [9].

Collagen, which is one of the main components of bone and cartilage, has been used in combination with chitosan in bone repair, and it has been demonstrated that using chitosan as the hydrogel could enhance osteogenesis and facilitate cellular proliferation and adhesion [30, 31]. The combination of chitosan with hydroxyapatite and gelatin provides a scaffold with compressive strength, which is essential for regeneration of trabecular bone [30]. Similarly, adding combined calcium phosphate cement (CPC) with chitosan results in increased mechanical strength and stability of the scaffold and induced osteogenic differentiation [32]. Using poly (lactic‐co‐glycolic) acid and BMP microspheres alongside of a chitosan scaffold with collagen results in the sustained release of integrated BMP and increased osteoconductive potentials [30]. Moreover, the combination of chitosan microspheres with alendronate and poly‐lactic acid and nanoparticle hydroxyapatite resulted in increased adhesion of cells via the microspheres and increased osteogenesis [30]. Similarly, using chitosan and silk fibroin resulted in increased cell adhesion and cellular migration and proliferation [30]. Regarding such unique properties of chitosan and the regenerative potential of stem cells, the combination of different scaffolds containing chitosan and stem cells is now becoming an area of interest in the field of bone and cartilage tissue regeneration. The following section focusses on the studies evaluating the efficacy of using chitosan‐containing scaffolds in delivering stem cells to damaged bone and cartilage tissues.

### 2.1 Potential of chitosan/collagen scaffolds in MSC‐mediated orthopaedic tissue engineering

Type 1 collagen is the main component of bone and essential for mineralisation [33]. Collagen enhances osteoblastic mineralisation, differentiation and adhesion [33]. There are reports showing that a combination of chitosan and collagen can provide favourable properties of each material for improving the potential of MSCs in tissue engineering. Maintenance of high cell viability during and following the encapsulation process is critical to ensure that the embedded cells can engraft and function at the repair site. The composition and properties of the matrix surrounding the cells can impact both their survival and function. Of note, it was shown that MSCs encapsulated in the 3D microbead matrixes with chitosan/collagen ratios of 35/65 and 50/50 clearly interacted with and spread inside the microbead matrix and kept high cell viability [34]. However, MSCs encapsulated in 65/35 microbeads retained a rounded morphology and a significant reduction in cell viability, suggesting that microbeads with higher collagen content are more conducive to the spreading of cells [34].

There is also evidence that shows chitosan/collagen microbeads can present an osteogenic stimulus to MSCs. Wise et al. used 3D collagen/chitosan hydrogel microbeads to encapsulate different populations of BM‐MSCs and compared the chondrogenic and osteogenic capacity of these cells [35]. After 21 days, the cells produced a considerable amount of OC and increased calcium deposition and phosphate mineralisation. Of note, either freshly isolated rat BM‐MSCs or purified and culture‐expanded rat BM‐MSCs encapsulated in hydrogel microbeads had a similar degree of osteogenesis; however, there were no signs of chondrogenic differentiation [35]. In the other study, Mathews et al. evaluated the effect of coating of the tissue culture plate with a tripolymer consisting of chitosan, collagen type 1, and hyaluronic acid on osteogenic differentiation of human BM‐MSCs. Hyaluronic acid is another major component of the extracellular matrix inducing migration and proliferation of MSCs. Notably, it was indicated that the tripolymer coating elevated mineralisation (calcium deposition), adhesion, and proliferation compared to the untreated or individual polymer‐coated plates. The results also showed that the tripolymer‐coated plate having a 1:1 mixing ratio of chitosan and type I collagen, as well as a surface modified with hyaluronic acid, is ideal for osteogenic differentiation of BM‐MSCs [33]. The transcription factor OSX is highly specific to osteoblastic differentiation and bone formation, and MSCs encapsulated in chitosan/collagen composite microbeads expressed a markedly high level of OSX gene, supporting the osteoinductive property of chitosan/collagen composite materials [34]. However, despite the increased expression of OSX genes, the expression levels of other markers of the osteogenic lineage, such as OC and bone sialoprotein (BSP) genes, were not significantly changed, showing the role of the OSX transcription factor in the molecular mechanisms behind the osteogenic properties of chitosan/collagen microbeads [34].

### 2.2 Potential of chitosan/CPC scaffolds in MSC‐mediated orthopaedic tissue engineering

CPCs are injectable scaffolds that are able to be reabsorbed and replaced by a new bone. CPCs have been approved by Food and Drug Administration for repairing craniofacial defects [36, 37].

Although CPCs are biocompatible and osteoconductive materials for use in orthopaedic tissue repair, slow integration with adjacent bone tissue is a major limitation for their use. Another possible limitation of CPC applications is the ionic activity and pH changes during the CPC setting reaction, which may negatively impact cells seeded directly on CPCs. Moreover, a lack of macropores in CPC limits the ingrowth of the new bone. There have been studies that show that the combination of CPCs with chitosan can enhance the flexural strength and increase adhesion and proliferation of stem cells, thereby improving the efficacy of CPCs in repairing bone defects [38].

Liu et al. showed that the chitosan/β‐glycerophosphate (GP) hydrogel could protect the MSCs during the CPC scaffold mixing, setting, and formation. Notably, chitosan/GP‐encapsulated MSCs were found to be adhered to the surfaces of CPC macropores, remain viable and normal, produce a considerable amounts of calcified nodules, and differentiate to osteogenic lineage [38]. This finding has been further supported by other studies that showed the effect of the combination of chitosan and various CPC formulations on MSC proliferation and differentiation. Zhao et al. used tetracalcium phosphate (TTCP) to fabricate a chitosan/CPC scaffold and showed that the human umbilical MSCs encapsulated in the chitosan/TTCP microbeads maintained marked viability, successfully differentiated into the osteogenic lineage, and synthesised bone minerals. This was accompanied with the increased expression of collagen I, OSX, OC, and ALP over 2 weeks of encapsulation in microbeads [39]. Similarly, Moreau et al. reported that the chitosan/TTCP composite scaffold could elevate the BM‐MSCs proliferation and attachment. It was also shown that the addition of chitosan to TTCP increased the ALP activity and differentiation to osteogenic lineage [36].

In the other study, Chen et al. investigated human embryonic stem cells (hE‐MSCs) seeded on CPC/chitosan scaffold immobilised with RGD [40]. It has been demonstrated that RGD‐bonded chitosan is more viscous and stickier than chitosan alone, which makes it more likely to bind to CPC. Tripeptide RGD can be recognised by adhesion receptors, improving immobilisation and mimicking the extracellular matrix. Chen et al. showed that hE‐MSCs could be successfully and highly seeded on RGD‐chitosan/CPC scaffolds with a higher immobilisation rate and higher strength and toughness than the chitosan/CPC without RGD [40]. The hESCd‐MSCs were found to have a high percentage of live cell density, mineralisation, and osteogenic differentiation on the RGD‐chitosan/CPC scaffold. Further studies showed that hE‐MSCs with a healthy spreading morphology are adherent to CPC, which expressed high levels of osteogenic markers, including ALP, OC, collagen I, and Runx2. Importantly, the mineral synthesis by hE‐MSCs on the RGD‐chitosan/CPC scaffold was twice that on the chitosan/CPC without RGD [40].

In addition, the mechanical property of the scaffold is another critical feature that must be considered for bone tissue engineering applications. Scaffolds need good mechanical properties, especially compressive modulus, to maintain their shape during the culturing and surgical procedures of transplantation [41]. Because scaffolds made from pure chitosan are soft and weak, they may not provide the necessary mechanical properties during in vitro and in vivo assays. Using CPCs as a reinforcing material can solve this problem [41]. Mohammadi et al. constructed a chitosan/CPC‐based hybrid 3D scaffold, in which the pores of the poly(L‐lacticacid) (PLLA)/β‐TCP matrix were filled with a biopolymer solution containing chitosan/gelatin/β‐TCP [41]. The skeletal PLLA/β‐TCP matrix provided the PLLA/chitosan/gelatin/β‐TCP with good mechanical properties and easy formation into desired shapes, while the chitosan/gelatin/β‐TCP microsponges gave the scaffold good cell interaction as well as a good degree of wettability for cell seeding. Moreover, since gelatin and chitosan have chemical structures similar to the repeating units of GAG, these elements were used as substitutes of GAG and collagen in the extracellular matrix. In vitro studies indicated the ability of cultured BM‐MCSs to be well‐attached, penetrated into the scaffold, and uniformly distributed. Notably, the expression of early and late phenotypic markers of osteoblastic differentiation, including ALP, OC, OP, and BSP, was upregulated in the scaffolds cultured in the osteogenic medium, and BM‐MCSs were found to self‐renew and differentiate into lineage‐committed osteoblast‐like cells forming mineralised nodules on these biocompatible and biodegradable scaffolds [41].

On the other hand, pure chitosan can be applied only to non‐weight‐bearing bone and cartilage defects due to its biomechanical properties. Notably, a combination of chitosan with biphasic calcium phosphate (BCP), a compound of hydroxyapatite and β‐tricalcium phosphate (β‐TCP), was found to be a promising osteoconductive candidate for repairing weight‐bearing bone defects. Liu et al. constructed a chitosan/BCP scaffold for delivering human urine‐derived stem cells (hU‐MSCs) and showed that hU‐MSCs can differentiate into osteoblasts, and the hUSCs adhered, proliferated, and differentiated on the constructed scaffold. Importantly, in vivo study indicated that the combination of hU‐MSCs and the chitosan/BCP scaffold could significantly promote bone regeneration more effectively compared with conventional pure chitosan or BCP scaffolds in large segmental bone defects in rabbits [42].

To sum up, the MSC‐laden chitosan/CPC hybrid scaffolds are promising to repair bone defects and improve bone tissue regeneration in orthopaedic and craniofacial applications.

### 2.3 Potential of other chitosan‐based hybrid scaffolds

Pure titanium and titanium alloys are commonly used orthopaedic implants due to their excellent biocompatibility and mechanical properties. Appropriate surface treatment of these alloys can greatly improve cellular attachment as well as interactions between implants and tissue. Chung et al. prepared a polyelectrolyte multilayer (PEM) coating containing chitosan and gamma‐poly[glutamic acid] (γ‐PGA) on a Ti6Al4V substrate, which facilitated the loading of BMP‐2 [43]. In vitro studies using rat BM‐MSCs showed that the chitosan/γ‐PGA scaffold is highly stable and biocompatible and does not exert any adverse effects on BM‐MSCs [43]. Chitosan/γ‐PGA PEM coating was found to be highly effective in carrying and enabling the staining release of BMP‐2, which promotes the morphogenesis of cartilage and bone tissue [43]. Such a coating induced osteogenic differentiation, as indicated by the increased ALP activity, and improved cellular mineralisation as indicated by the increased calcium content [43]. Yang et al. considered the chitosan‐modified poly[L‐Lactide‐co‐Epsilon‐Caprolactone] (PLCL) scaffold for delivering human BM‐MSCs [44]. PLCLs are elastic biomaterials with hydrophobic nature without cell recognition sites, which are their main drawbacks. To overcome this limitation, the authors cross‐linked de‐acetylation chitosan to PLCL by an aminolysis approach. Using the straightforward chemically cross‐linking of chitosan on the surface of the PLCL scaffold, the surface roughness and the surface hydrophilicity of the PLCL scaffold were increased without changing the porosity of scaffold porosity [44]. When BM‐MSCs were seeded on the chitosan‐modified PLCL, the chitosan surface induced a more consistent and even distribution of the seeded MSCs within the scaffold. Notably, BM‐MSCs rapidly adopted a distinct spread‐up morphology on attachment on the chitosan‐modified PLCL scaffold with the formation of F‐actin stress fibres providing better cell aggregation. The cartilage formation on the chitosan‐modified PLCL was also found to be highly enhanced; the chitosan modification of the PLCL scaffold could improve the cell compatibility of the PLCL scaffold without a significant change of the physical elastomeric properties of PLCL and led to the cartilage tissue formation with a high quality [44].

## 3 THE ROLE OF CHITOSAN‐BASED SCAFFOLDS IN MSC‐MEDIATED ORTHOPEDIC TISSUE ENGINEERING

Chitosan has high osteocoductivity, porosity, and appropriate pore size distribution, which is a good choice for loading of stem cells and a homing place for proliferated bone tissue [9]. As discussed in the following sections, chitosan has the potential to act as an osteoconductive as well as a delivery system regarding MSCs in orthopaedic tissue engineering.

### 3.1 The synergistic effects of chitosan on osteogenic differentiation of MSCs

The effect of chitosan on self‐renewal and proliferative potential of stem cells has been reported and it has been demonstrated that MSCs form 3D spheroids on the chitosan membrane and their stemness markers, such as Sox2, Oct2, and Nanog, remain either unchanged or upregulated [45]. Moreover, chitosan can increase the homing of circulating stem cells in the body and increase tissue healing as previously described by Liu et al. [46]. They demonstrated that chitosan hydrogel can improve the myocardial infarction environment and enhance stem cells engraftment and homing [46]. Similarly, chitosan‐based scaffolds have a synergic effect on stem cell regenerative potential in bone tissue repair. Liu et al. demonstrated the synergic effect of using stem cells and chitosan‐based scaffolds as the increased rate of degradation of chitosan and tissue regeneration [42]. They showed that the rapid degradation of chitosan scaffold and bone differentiation and regeneration induced by stem cells create a degradation and regeneration cycle, which is suitable for achieving strengthened bone tissue in shorter time [42]. The synergic effects of using chitosan‐based scaffolds on MSC proliferation and differentiation have been addressed in other studies as well. Moreau et al. and Mohammadi et al. studies evaluating the effect of CPC‐chitosan and PLLA/chitosan/gelatin/β‐TCP hybrid scaffolds, respectively, demonstrated the synergic effect of these scaffolds on MSCs by revealing the increased ALP level as a marker of stem cell differentiation to bone tissue [36, 41]. The study by Chen et al. showed that CPC‐chitosan‐RGD scaffolds provided considerable attachment for stem cells and therefore increased their final concentration after transplantation [40]. This study demonstrated that CPC‐chitosan‐RGD scaffolds increased the expression of ALP, collagen type I, OC, and Runx2 genes, indicating the synergic effect of this scaffold on stem cells [40]. Similarly, the Zhao et al. study using preosteodifferentiated MSCs encapsulated with CPC scaffolds has a synergic effect on increasing the expression of ALP, OC, collagen type I, and osterix [39]. A study by Chung et al. study revealed that, considering chitosan/γ‐PGA, PEM coating can induce a gradual release of BMP‐2, which further triggered the early differentiation of MSCs loaded on this scaffold [43]. The Wang et al. study evaluating the potential of chitosan/collagen microbeads contacting MSCs demonstrated that chitosan/collagen materials have osteoinductive potentials on MSCs [34]. Mathews et al. reported that the collagen and HA used in their chitosan containing scaffolds can have a synergic effects on stem cells increasing their differentiation and bone mineralisation [33]. A study by Yang et al. demonstrated that considering a hydrophic chitosan modified scaffolds as PLCL scaffolds with chitosan coating increased the synergic effect of stem cells by increasing their adhesion, differentiation, and cytoskeletal organisation [44].

### 3.2 Chitosan as a delivery vehicle for MSCs

**FIGURE 2:**
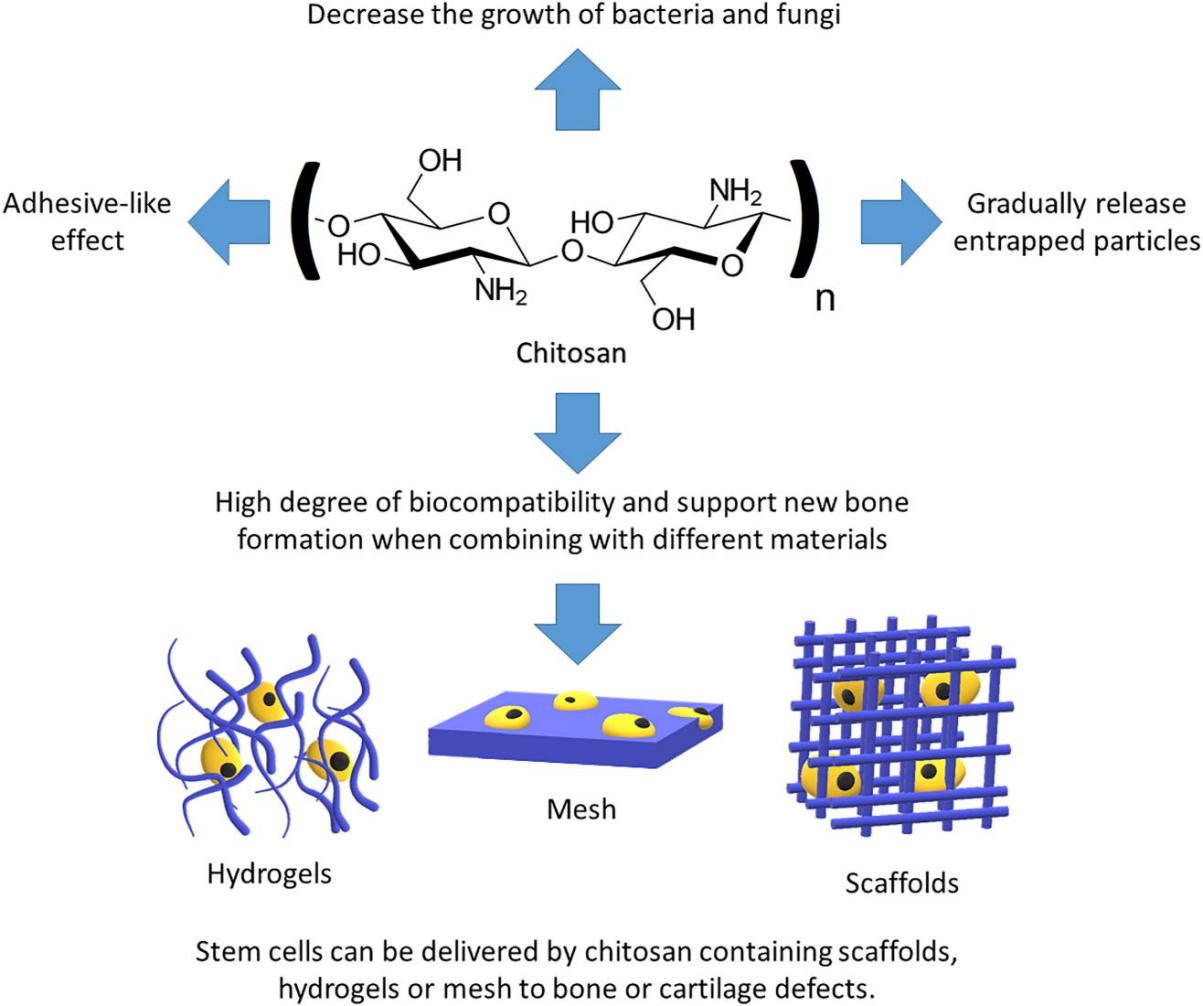
Chitosan‐based materials have adhesive‐like behaviours and can gradually release entrapped particles. Moreover, antifungal and antibacterial effects of chitosan made this material a good choice for medical use. Considerable biocompatibility of chitosan in combination with different types of mesh, scaffolds, or hydrogels is used in stem cell‐based therapies in bone and cartilage tissue regeneration

Chitosan‐based delivery systems have been previously used in different diseases and tissue regeneration. The incorporation of stem cells and other biomolecules into chitosan‐containing scaffolds is the most common strategy for enhancing regenerative therapies for different tissues [47]. The most important advantage of using chitosan in tissue regeneration is its adhesiveness to stem cells [11]. Chitosan scaffolds are an appropriate delivery system for stem cells as these cells can adhere to chitosan either located at the surface of the scaffold or at the pores located within the biomaterials [3]. Such adhesive potential lets the stem cells proliferate at the surface of the scaffold, and when the scaffold degrades gradually, these cells maintain their high density and provide trabecular shapes strong enough for bearing weight (Figure 2) [10]. Similar to direct engraftment of chitosan‐containing scaffolds in damaged tissues, chitosan microbeads are the other form of the chitosan delivery system, which can deliver stem cells to the desired tissue even by using small needles [34]. Stem cells can be encapsulated with chitosan and the collagen matrix, protecting them for efficient delivery to damaged bone and cartilage tissue [34]. Chitosan nanoparticles play an important role in stem cell research and cell reprogramming. Many functional proteins essential for reprograming of cells, including OCT4, SOX2, and c‐MYC, can be delivered to cells via chitosan nanoparticles [48]. Similarly, chitosan nanoparticles loaded with specific microRNAs, including has‐miR‐199a‐5p, could induce osteogenic differentiation of human MSCs [49]. Therefore, these nanoparticles are considered as an efficient tool for generating transgene‐free MSCs. Moreover, modified chitosan nanoparticles with specific materials, such as gold, have been reported to induce osteogenic differentiation of adipose‐derived MSCs [50].

## 4 CONCLUSION

Chitosan is a copolymer of glucosamine and N‐acetylglucosamine containing HA and GAG. The presence of HA and GAG makes chitosan resembling cartilage tissue, which is mainly used for the treatment of cartilage injury, and in the combination of chitosan with other scaffold molecules, including collagen type I converts chitosan to a potent scaffold for treating bone lesions. Although using chitosan as a scaffold for treating bone and cartilage tissue has promising results for treating orthopaedic diseases, available evidence suggests that using the combination of chitosan‐based scaffolds with MSCs can improve the healing of bone and cartilage tissue lesions. Different forms of chitosan‐based scaffolds can provide a potent homing environment for MSCs and facilitate their proliferation and differentiation to bone and cartilage tissue. Moreover, other potentials of using chitosan‐based materials in regenerative medicine should be considered for further tissue research. These potentials are mainly based on the effect of chitosan on blood coagulation and antibacterial‐antifungal effects of this material, which made it a good choice for tissue dressing.

## AUTHOR CONTRIBUTIONS

Xiuju Zhang contributed to the conception and design of the study. Ping Qi and Zhaohui Ning performed data acquisition and prepared the first draft. Xiuju Zhang revised the draft critically. All authors read and approved the final version to be submitted.

## CONFLICT OF INTEREST

The authors declare that there are no conflicts of interest and financial support for the present review article.

## PERMISSION TO REPRODUCE MATERIALS FROM OTHER SOURCES

None.

## Data Availability

Data sharing is not applicable to this article as no datasets were generated or analysed during the current study.
